# Demography, patterns of care, and survival outcomes in patients with salivary duct carcinoma: an individual patient data analysis of 857 patients

**DOI:** 10.2144/fsoa-2021-0052

**Published:** 2022-03-08

**Authors:** Prashanth Giridhar, Bhanu Prasad Venkatesulu, Ryan Yoo, Pragathee V, Goura K Rath, Supriya Mallick, Ashishdutt Upadhyay, Dennis Pai Chan

**Affiliations:** 1 Department of Radiation Oncology, Tata Medical Centre,Varanasi; 2 Loyola University Chicago, Loyola University Medical Center &/or Edward Hines Jr., VA Hospital, Stritch School of Medicine, Department of Radiation Oncology, Cardinal Bernardin Cancer Center, Maywood, IL 60153, USA; 3 Department of Internal Medicine, Karpagam Faculty of Medical Sciences & Research, Coimbatore, Tamil Nadu, India; 4 Department of Radiation Oncology, All India institute of medical sciences, New Delhi, India; 5 Department of Biostatics, All India Institute of Medical Sciences, New Delhi, India

**Keywords:** adjuvant, androgen deprivation therapy, androgen receptor, carcinoma, Her2/neu overexpression, parotid, patterns of care, radiotherapy, salivary duct, surgery

## Abstract

**Aim:**

Salivary duct carcinoma (SDC) is a rare and aggressive malignancy. The optimal treatment protocols are debated.

**Methodology:**

A systematic search and individual patient data analysis of published cases of SDC was performed. SPSS v21 was used for statistical analysis.

**Results:**

Data of 857 patients available. Median overall survival (OS) and progression-free survival (PFS) of the entire cohort 42 months and 24 months. Nodal involvement, males, primary size >5 cm, androgen receptor (AR) negativity significantly worse OS. Patients with surgery had a favorable median PFS (p = 0.000) and OS (p = 0.077). Patients with adjuvant radiation had better PFS (30 vs 18 months; p = 0.077).

**Conclusion:**

SDC has modest survival. Surgery and adjuvant radiation should be advocated for all patients. AR expression appears prognostic for survival.

## Plain language summary

Salivary duct cancer (SDC) is a rare cancer. The exact care choices are debated. All published cases of SDC were pooled and analysis was carried out. Data from 857 patients were available. Median survival of the entire cohort was 42 months. Nodal involvement, males, large tumour, androgen receptor (AR) negativity worsened survival. Patients who underwent operation and radiation were better able to take care of illness. SDC has modest survival. Operation and radiation should be made available for all patients. AR expression changes survival results.

Salivary duct carcinoma (SDC) is an aggressive malignancy of the ducts of salivary gland that closely mimics high-grade breast ductal carcinoma. It accounts for 2% of salivary gland tumors with high propensity for local recurrence and distant metastases [1]. It is predominately a disease of male gender at sixth decade of life. SDC is characterized by increased expression of androgen receptor (AR), amplification of ERBB2 (Her2/neu pathway), PI3K mutations, etc. [2]. Current standard of care is surgery followed by radiation. However, indications for radiation in the present era of molecular targeted therapy, impact of AR and Her2/neu expression on prognosis is not yet established. The high propensity for distant metastasis also calls into question whether SDC should be considered a systemic disease with integration of targeted therapies and/or systemic chemotherapy in addition to local therapeutic modalities like surgery and radiation. A systematic approach to pool the available literature and understanding the patterns of care may lead to new insights and may optimize the management of SDC. We conducted this systematic review and individual patient data analysis of SDC with an aim to understand patterns of care, impact of AR and Her2/neu expression on survival, role and indications of adjuvant radiation, chemotherapy and androgen deprivation therapy.

## Search methodology

### Literature search

The PubMed (National Institutes of Health) and Google scholar were searched with the key words – salivary duct carcinoma; salivary duct carcinoma AND radiotherapy; salivary duct carcinoma AND chemotherapy; salivary duct carcinoma AND trastuzumab; salivary duct carcinoma AND androgen deprivation therapy; Stenson duct carcinoma; ‘parotid duct carcinoma’. We restricted our search to English language only and all publications till 1 April 2019 were considered eligible. The search spanned the duration from the inception of each database up to 1 April 2019.

Two authors independently searched the databases with application of search terms for the relevant articles and any disagreements were resolved by mutual discussion. The corresponding author was the point person for resolving any disagreement in the inclusion of studies. All three of the authors are trained radiation oncologists with knowledge of search protocols and previous experience in performing systematic reviews.

## Article review

**Figure 1.**
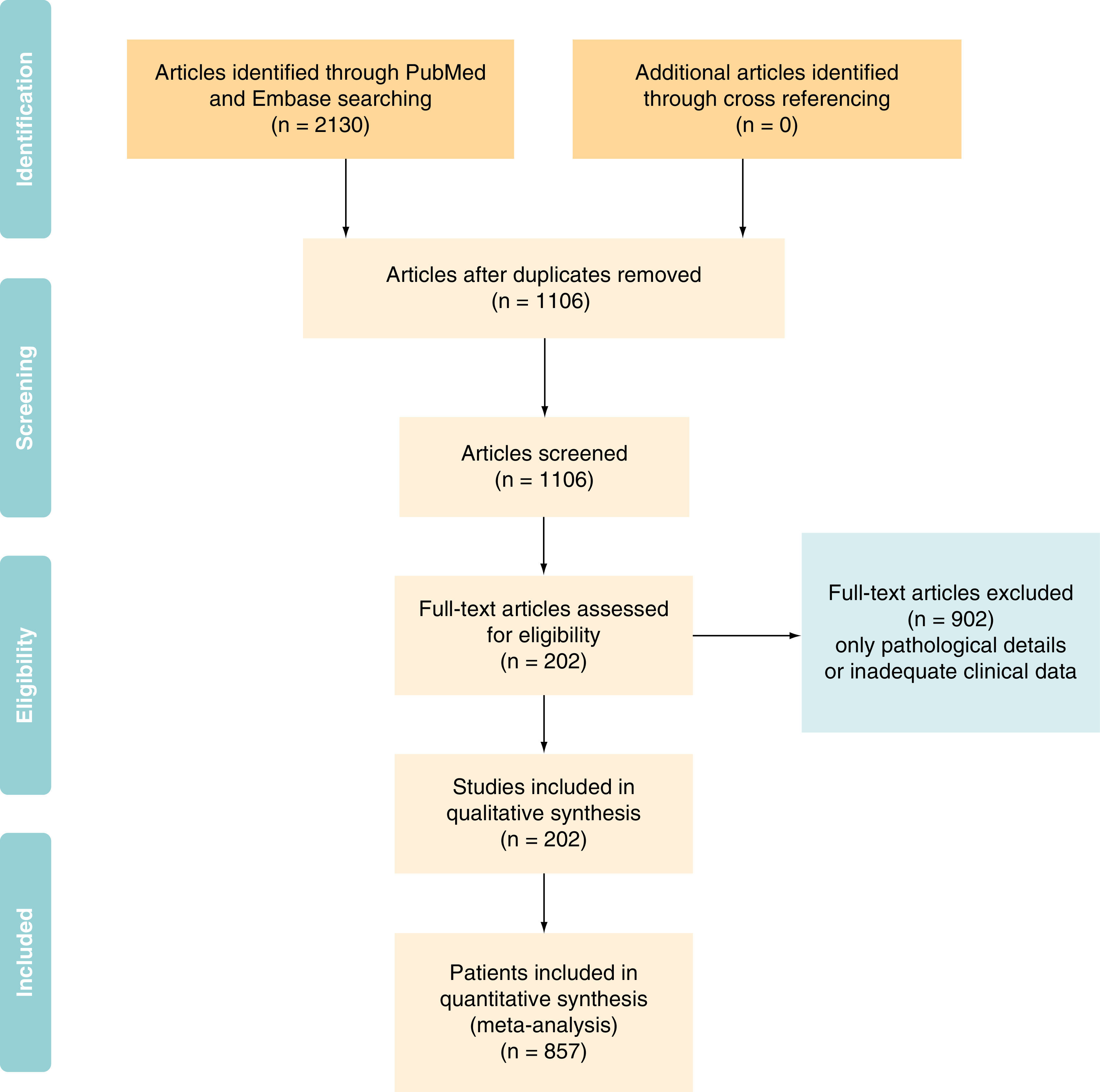
PRISMA diagram showing selection of patients for the individual patient data.

A systematic approach was followed by authors for reviewing the eligibility of articles. The articles from the initial search of the electronic databases were imported into reference manager software. An independent review of the abstracts and full paper articles was done. The duplicates were removed, and the titles of articles were evaluated. Abstracts found to be relevant to the topic of interest were shortlisted. Then the full-length paper of the shortlisted articles was assessed for the eligibility criteria. Inclusion criteria: patients with histo-pathological diagnosis of salivary duct carcinoma with atleast one or more clinical features available. This included articles where in some clinical and demographic features were described but treatment was not described. Exclusion criteria: Articles with no clear histological proof of SDC and articles with only pathological description available. On completion of search, following information was tabulated in the predesigned excel chart – sex, *de novo* or secondary carcinoma, location, size of primary, clinical nodal involvement, metastatic disease at presentation if any, pathological information (pathological lymph node status, androgen receptor expression, Her2/neu over expression, PI3K mutation), adjuvant treatment received, duration of progression-free survival (PFS), overall survival (OS), site of recurrence and salvage treatment. Preferred Reporting Items for Systematic Reviews and Meta-Analyses (PRISMA [Figure 1]) explains the data synthesis from the eligible studies.

## Statistical analysis

Data was analyzed and categorical variables were summarized by frequency and percentage and quantitative variables by the median and range. PFS and OS were calculated from the date of diagnosis to the date of documented progression and death, respectively. Chi square test was used to identify selection bias of candidates for different variables. Kaplan–Meier method was used for survival analysis. Univariate analysis using log rank test was done to find the impact of age, gender, *de novo* versus ex-pleomorphic adenoma, location, size of primary, nodal status, surgery, lymph node dissection, androgen receptor expression status, Her2/neu status, use of radiation, use of chemotherapy, androgen deprivation therapy and targeted therapy on PFS and OS was analyzed. Multivariate analysis with cox regression analysis was performed. A p value of <0.05 was taken as significant. SPSS v21 was used for all statistical analysis.

## Quality & quantity of data

All data retrieved were from case reports, case series and retrospective data analysis. A total of 857 cases were suitable for statistical analysis. Survival status (i.e, alive or deceased) was available for 636 (74.2%) patients. Data regarding performance of surgery, radiotherapy and chemotherapy were available for 732 (85.4%), 579 (67.6%) and 261 (30.5%) patients, respectively. Data regarding completeness of resection of primary was clearly described in only 28 (3.8%) patients. For our analysis, case reports mentioning 3+ on immunohistochemistry (IHC) or Her2/neu amplification were coded as positive. Any report reporting 2 or less on IHC or Her-2-neu negative was coded as negative. For our analysis regarding androgen receptor, articles describing IHC as negative or <10% cells showing positivity were coded AR negative while articles describing IHC as positive or >10% cells positive were coded AR positive.

## Results

### Patient characteristics

**Figure 2.**
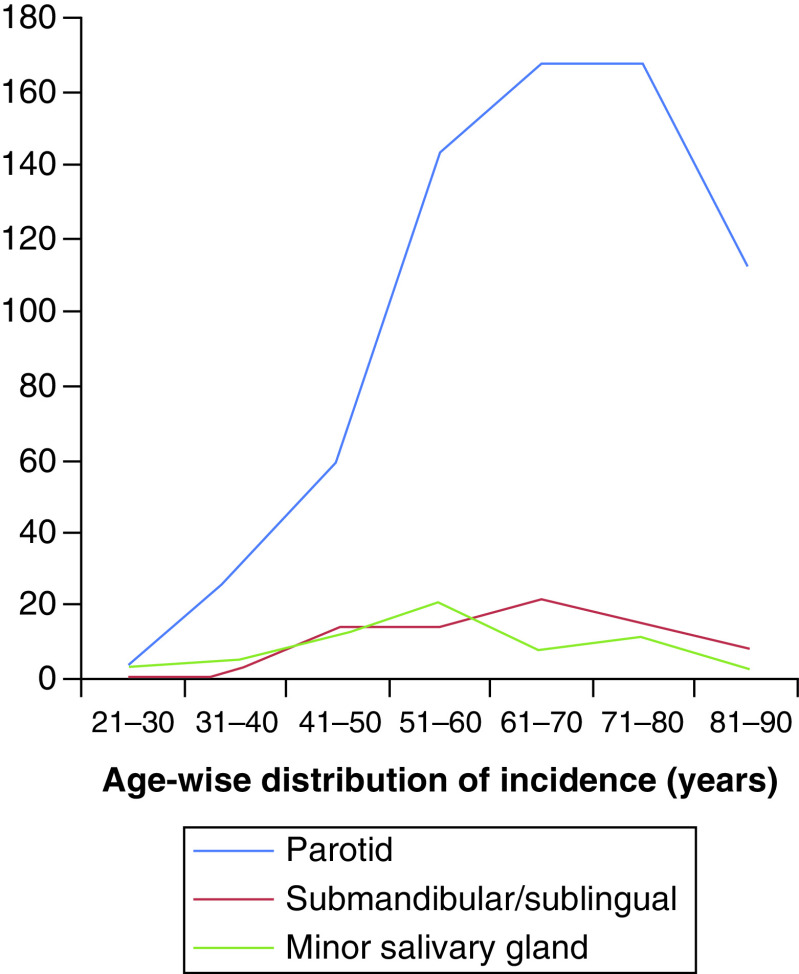
Incidence of salivary duct carcinoma according to different age groups and different primary subsites.

A total of 857 patients were included in the analysis. There was a clear male preponderance with a male to female ratio of 3.2: 1. Median age of presentation was 63 years (range: 22–91 years). SDC, secondary to pleomorphic adenoma was documented in 108 (12.6%) patients. Parotid was the most common site of primary (n = 631; 73.6%) followed by submandibular glands (n = 85; 9.9%). Figure 2 shows location of primary site age distribution. Information on clinical nodal involvement was available in 217 (25.3%) patients. Of the 217 patients, 127 (58.5%) patients were clinically node positive. Of all the cases, only 20 (2.3%) patients presented with a distant metastatic disease. Information on pathological lymph node status was available in 143 (16.7%) patients of whom 97 (67.8%) were node positive. AR status was available in 146 (17%) cases of which 125 (85.6%) were AR positive. Information regarding Her2/neu over expression was available for 153 (17.9%) patients and 71 (46.4%) patients had documented Her2/neu over expression. Other common mutations documented were PI3K (n = 9), p53 (n = 7), c-kit (n = 1), PTEN (n = 1) and RET (n = 1).

### Treatment characteristics

Information on surgery was documented in 732 (85.4%) patients of whom 709 (96.9%) patients underwent surgery. In addition to surgery for the primary 357 (50.5%) patients underwent lymph node dissection. Interestingly, ten patients with distant metastatic disease also underwent surgery. 23 patients did not undergo surgery. The reasons for inoperability were metastatic disease at presentation (n = 10), large bulky nodes (n = 1), fungating mass (n = 7), cavernous sinus involvement (n = 1), multiple comorbidities (n = 1) and patient refusal (n = 3). 26 patients had a documented positive or close margin. Data regarding radiotherapy was available in 579 (67.6%) patients. 422 (72.9%) patients underwent surgery and adjuvant radiotherapy (with or without chemotherapy) while 127 patients underwent surgery only. Surgery and adjuvant chemotherapy (without radiotherapy) was given in five patients only. The analysis presented in the manuscript is based on univariate analysis since multivariate analysis was not significant. Details regarding patient demography and treatment characteristics are summarized in Table 1.

**Table 1.** Demographic and treatment characteristics of patients with salivary duct carcinoma.

Demographic features	Patients (n)	Percentage
Sex Male Female Information not available	597 188 72	69.7 21.9 8.4
History of pleomorphic adenoma Yes No Information not available	108 54 695	12.6 6.3 81.1
Location of primary Parotid Submandibular gland Minor salivary gland Others Information not available	631 85 44 9 88	73.6 9.9 5.2 1.1 10.2
Size of primary <5 cm >5 cm Information not available	321 51 485	37.5 6 56.5
Clinical nodal involvement Yes No Information not available	127 90 640	14.8 10.5 74.7
Pathological nodal involvement Yes No Information not available	97 49 711	11.3 5.7 83
Androgen receptor status Positive Negative Information not available	125 21 711	14.6 2.4 83
Her2/neu over expression Yes No Information not available	71 82 704	8.3 9.7 83

**Treatment characteristics**		
Surgery Yes No Information not available	709 23 125	82.7 2.7 14.6
Nodal dissection Yes No	357 352	50.5 49.5
Adjuvant radiotherapy Yes No Information not available	422 157 278	49.2 18.3 32.5

### Survival analysis

Median OS and PFS of the entire cohort were 42 (95% CI: 34.6–49.3) and 24 months (95% CI: 17.7–30.2), respectively.

### Effect of clinical & pathological prognostic factors on survival

Male gender had a worse PFS compared with females (24 months; 95% CI: 18–29 vs 36 months; 95% CI: 19–52 months; p = 0.05). Patients presenting with a large primary (size >5 vs <5 cm), clinically node-positive disease and Her2/neu over expression had numerically worse PFS (12 vs 32 months; p = 0.583); (12 vs 16 months; p = 0.560); (14 vs 24 months; p = 0.121), respectively. Patients with tumors that expressed AR had a numerically better PFS in comparison to patients who did not express it (32 months; 95% CI: 0–60 months) versus 15 months; 95% CI: 0–not reached; p = 0.500).

Patients presenting with a larger primary (size >5 vs <5 cm) had significantly worse OS (28 months; 95% CI: 16–39 vs 51 months; 95% CI: 33–69 months; p = 0.014). Clinical and pathological node positivity at presentation conferred worse OS (26 vs 110 months; p = 0.000) and (33 versus 110 months; p = 0.008), respectively. AR-positive patients had significantly favorable OS (39 months; 95% CI: 25–53 vs 31 months; 95% CI: 26–36 months; p = 0.023). Interestingly, Her2/neu over expression had no bearing on OS (p = 0.459). Due to low numbers, effect of AR status could not be assessed in multivariate analysis. Multivariate analysis of remaining factors suggested that pathological lymph node positivity remained a significant factor in affecting OS (p = 0.03).

### Effect of surgery on survival

Patients who underwent surgery had a favorable PFS and trend toward favorable OS compared with those treated with non-surgical approach (24 months; 95% CI: 16–32 versus 6 months; 95% CI: 2–10 months; p = 0.000) and (44 months; 95% CI: 35–52 months; p = 0.077), respectively. Patients who underwent nodal dissection showed no differences in PFS PFS (24 vs 17 months; p = 0.193) and showed in no difference in OS (36 vs 44 months; p = 0.096). Chi square test revealed that significantly higher number of patients with larger tumors (>5 cm) and clinical node positivity had been treated with nodal dissection. As only 38 patients underwent elective nodal dissection, meaningful analysis could not be performed.

### Effect of adjuvant radiotherapy on survival

**Figure 3.**
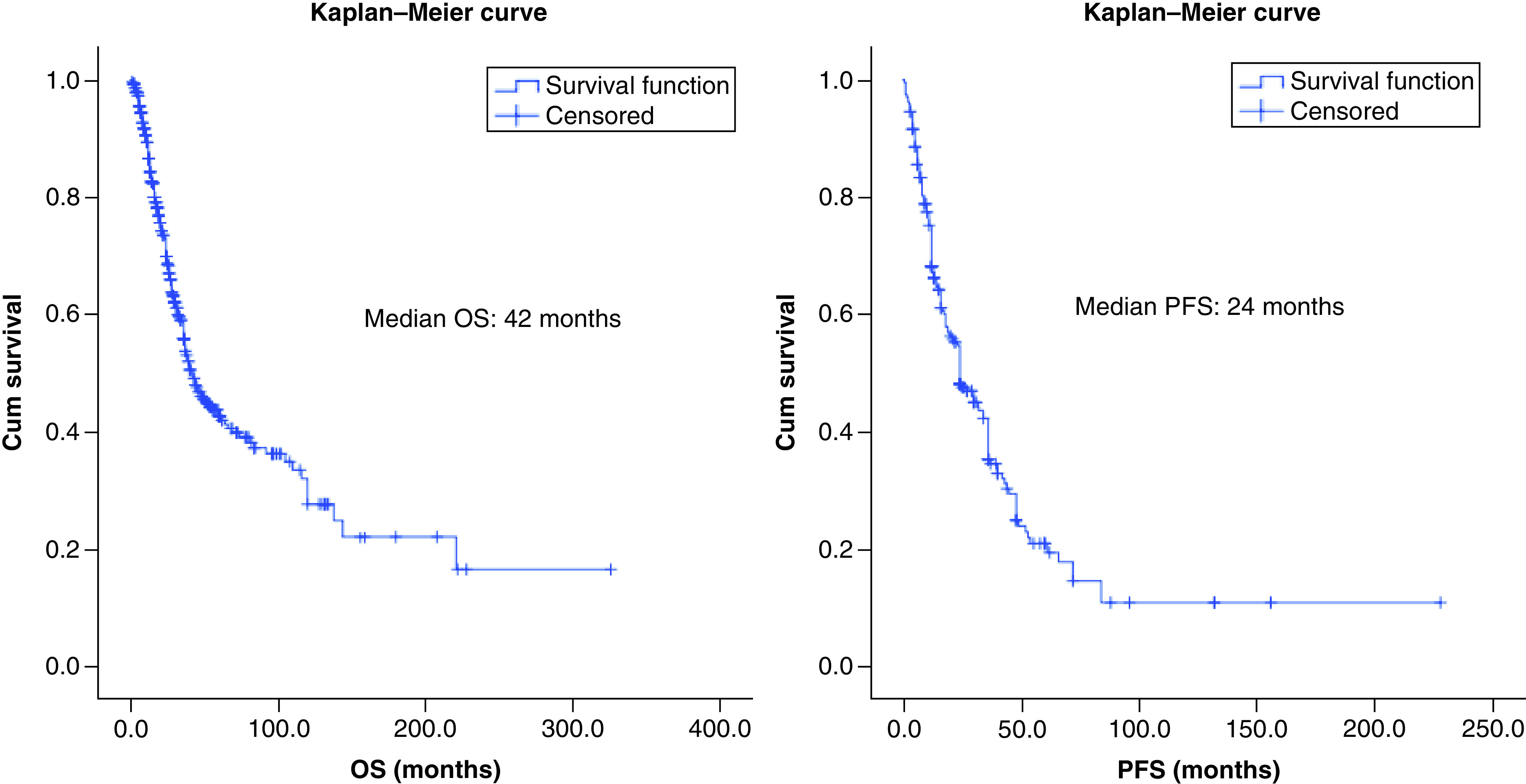
Kaplan–Meier survival graph showing overall survival and progression-free survival. OS: Overall survival; PFS: Progression-free survival.

**Figure 4.**
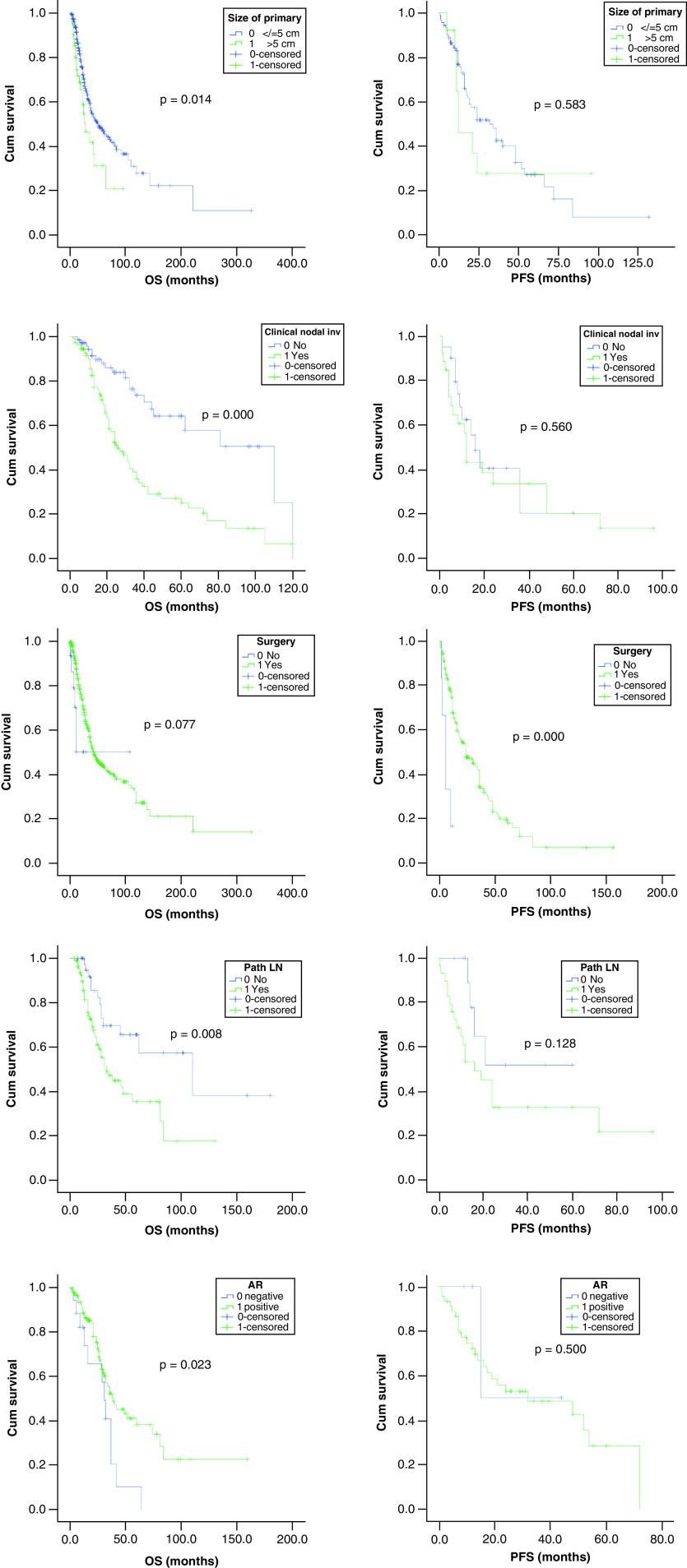
Kaplan–Meier survival graph showing impact of size of primary greater than 5 or less than 5 cm, clinical nodal involvement at presentation, surgery, pathological lymph node positivity, and AR positivity overall survival and progression-free survival. AR: Androgen receptor; OS: Overall survival; PFS: Progression-free survival.

**Figure 5.**
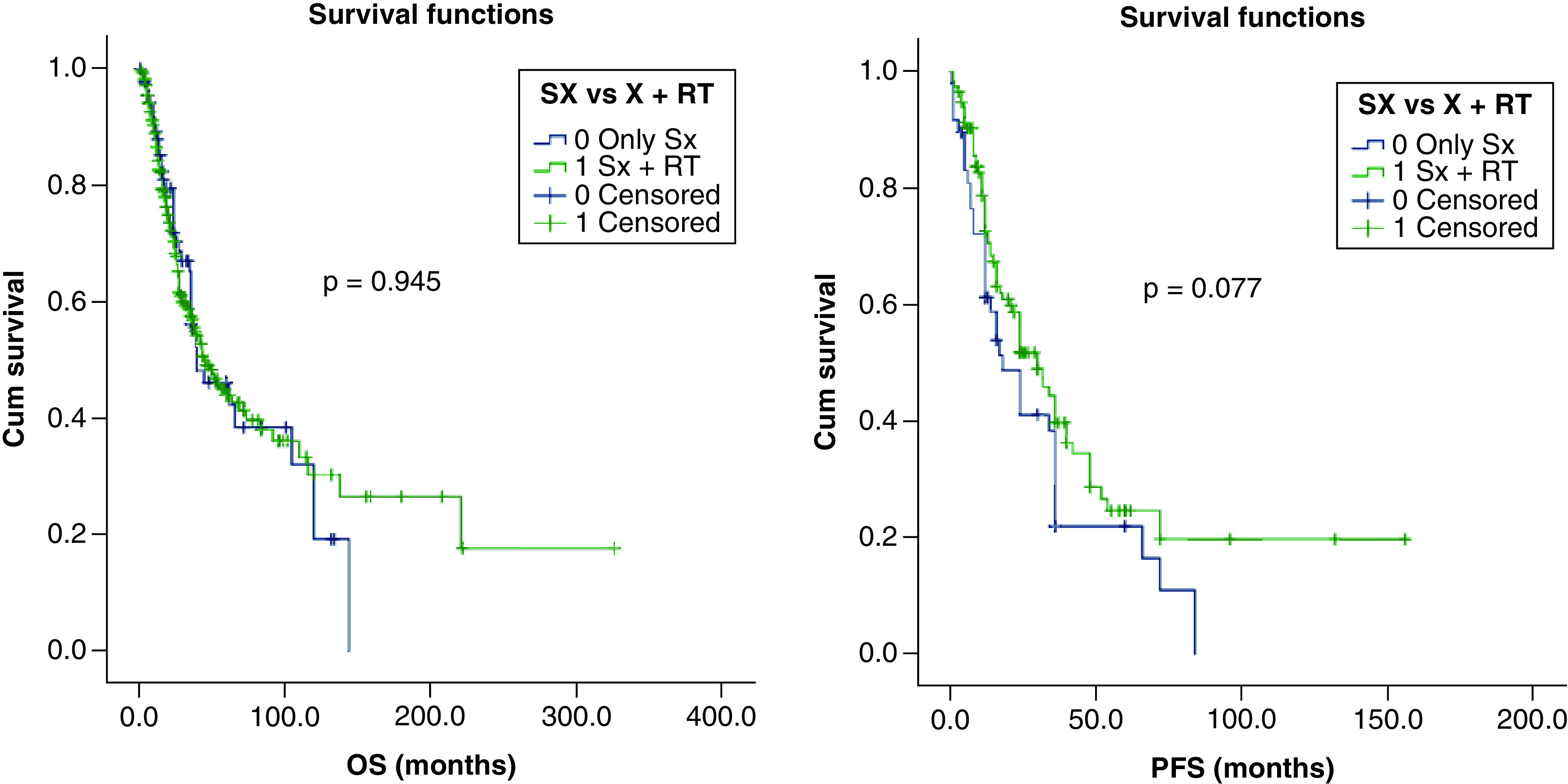
Kaplan–Meier survival graph showing impact of surgery versus surgery + adjuvant radiation, overall survival and progression-free survival. OS: Overall survival; PFS: Progression-free survival.

Due to lack of consensus and evidence, use of adjuvant radiotherapy was individualized in most case reports. We performed a chi square test to identify if selection bias of candidates for radiotherapy was present for the above identified prognostic factors of survival (sex, tumor size >5 cm, clinical and pathological nodal positivity and AR-negative status). The analysis revealed that significantly higher number of patients with clinical and pathological nodal positivity received radiotherapy. Patients who received adjuvant radiation versus no adjuvant radiation had better PFS (30 months; 95% CI: 21–38 vs 18 months; 95% CI: 10–26 months) with a trend toward statistical significance (p = 0.077). However, there was no such impact on OS (46 vs 40 months; p = 0.945). Figures 3–5 shows the Kaplan–Meier curves of the factors that have a bearing on PFS and OS.

### Effect of adjuvant chemotherapy & androgen deprivation therapy on survival

Surgery followed by adjuvant chemotherapy was employed in five patients. Median OS of this cohort was 21 months. No other meaningful analysis could be performed. Seventy-one (8.3%) patients received adjuvant conventional chemotherapy in addition to radiation in the primary treatment. Median OS of this cohort was 34 months (95% CI: 30–38 months). Due to low patient numbers, we restrained from performing further analysis. Androgen deprivation therapy was used in primary treatment in 33 patients. ADT did not have a significant effect on OS in univariate analysis (p = 0.773). Low patient number prevented us from analyzing its effect on PFS.

### Patterns of recurrence

Recurrence occurred in 282 (33%) patients in this cohort. Median time to distant recurrence was 10 months (range: 1–72 months) and 7 months (range: 0.5–48 months) for loco-regional recurrence. Site of recurrence was documented in 139 patients with 108 patients (77.7%) developing distant metastases while the rest had loco-regional recurrence (22.3%). The most common site of distant recurrence was the lung (n = 79; 9.2%) followed by bone (n = 50; 8.7%) and brain (n = 22).

## Discussion

This is the largest pooled study of SDC with 857 cases as part of the analysis. Median PFS and OS were found to be 24 and 42 months highlighting high aggressiveness. Aggressiveness of SDC could also be established from the fact that nearly 58% were node positive at diagnosis and pathological node positivity was prognostic of OS on multivariate analysis. Surgery was the single most common treatment modality advocated in all but 3.2% patients and closely resembles finding of other published series [3]. Patients who underwent surgery had a favorable PFS (24 months; 95% CI: 16–32 vs 6 months; 95% CI: 2–10 months; p = 0.000). It is important to note the inherent risk of bias with this finding as most patients who did not undergo surgery presented either with a higher stage of disease or were not medically fit for surgery. Patients who underwent nodal dissection had a trend toward improved PFS but poorer OS. The pathological node positivity has higher propensity for local and regional recurrence implies that more aggressive treatment approaches including adjuvant radiation and adjuvant systemic therapy based on molecular profile are needed. These findings provide strong basis for incorporating routine nodal dissection as part of management of SDC given the very high node positivity rates as well as prognostic importance of pathological nodal positivity. It is seen in our study that patients with poor prognostic factors like larger size primary and clinical node positivity underwent nodal dissection more commonly. This selection bias along with tendency of distant metastases probably negated the impact of nodal dissection on OS. A recent NCDB analysis of 8243 patients of salivary gland tumors concluded significantly better survival with adjuvant radiation in high-risk groups [4]. In the present analysis, a trend toward better PFS was seen in patients treated with adjuvant radiotherapy. It was also seen that selection bias was present while selecting patients for radiotherapy with patients with nodes more likely to have received treatment.

From the present study, interestingly AR positivity has emerged as a potential prognostic factor in SDC. For the purpose of the study, we assumed a cut off of 10% to assume AR negativity and positivity on IHC based on a lower threshold chosen in some previous reports [5]. 125 (85.6%) patients were AR positive which is comparable with 89–96% AR positivity shown in other published series [6–9]. Patients with AR-positive tumors had significantly better OS ([OS: 39 months; 95% CI: 25–53 months] – 31 months; [95% CI: 26–36 months] [p = 0.023]) compared with AR-negative tumors. In the present analysis, androgen deprivation therapy (ADT) was used in primary treatment with surgery for 33 patients only and it had no significant impact on OS in univariate analysis (p = 0.773). There is also no clear consensus on the duration of ADT in the adjuvant setting. In a recent retrospective analysis of adjuvant ADT for high risk of relapse (stage IV A) patients, ADT improved disease-free survival on multivariate analysis [10]. But the conclusions in that study were drawn from only 22 patients. A larger patient dataset is therefore required before any recommendation for adjuvant ADT may be made. Since SDC tends to have AR expression, androgen blockade is a potential tool as salvage in patients with recurrent disease. In a recent retrospective analysis by Viscuse *et al.* [11], it was seen that ADT provided better response rates than chemotherapy in recurrent/metastatic SDC with comparable OS. In another published analysis [12], ADT provided a clinical benefit rate of 50% in advanced SDC. ADT may therefore be preferred to chemotherapy in AR positive recurrent SDC. The other molecular perturbation that is commonly reported in SDC is HER2 amplification/over expression which ranges from 27 to 44% in agreement to 46% Her2/neu over expression in the present study [13,14]. In univariate analysis, patients with Her2/neu over expression appear to have a worse PFS (median PFS: 14 versus 24 months; p = 0.121). Interestingly, a recent phase II trial reported promising results in HER2-positive advanced unresectable SGC with a response rate of nearly 70% with trastuzumab and docetaxel [15]. Her2/neu-targeted therapy can be used a potential salvage treatment option for SDC patients. The role of anti-Her2/neu blockade as adjuvant therapy is worth exploring in select patients. Amini *et al.* in another recent NCDB analysis reported no advantage of adding chemotherapy along with adjuvant radiation in salivary gland carcinoma. In the present analysis, we refrained from analyzing the impact of concurrent chemotherapy as only 8.3% patient's received chemotherapy along with radiation [16].

As the patients with SDC have a moderate survival follow-up protocol is immensely important. Median time to locoregional recurrence is 7 months and distant metastasis 10 months. A closer follow-up with physical examination and contrast enhanced computed tomography (CT) scan of the face, neck and chest should be repeated every 3 months in the first 2 years. Subsequently, physical examination may be done every six monthly with annual CT of the face, neck and chest. A bone scan or CEMRI and only MRI without CE brain may be done in symptomatic patients. The above individual patient data (IPD) analysis is based on previously published case reports; case series and is limited by the inherent publication and selection bias. In addition, data may not be complete in all respect forcing the analysis of different parameters to the available sample size alone. Data of AR and Her2/neu is subjective in the absence of a central standardised analysis The IPD analysis by us is an pooled analysis of case reports and series published from different parts of the world and hence should be considered a true representation of disease incidence and survival outcome. We are reporting data of 857 patients which to our knowledge is the largest cohort on SDC and should be considered the strength of the analysis. We also attempted to look into the impact of AR and Her2/neu as prognostic and predictive factor with the largest sample size which should also be considered strength of the study.

## Conclusion

SDC is a disease with modest survival. Surgery is the cornerstone of therapy and adjuvant radiation should be advocated in all patients. Nearly 90% patients show AR positivity, but the role of ADT is yet to be established in the adjuvant treatment. Similarly, nearly 50% patients show Her2/neu over expression which appears to impart worse PFS and therefore merits exploration of targeted agents in the treatment of such patients.

## References

[1] Jayaprakash V, Merzianu M, Warren GW Survival rates and prognostic factors for infiltrating salivary duct carcinoma: analysis of 228 cases from the Surveillance, Epidemiology, and End Results database. Head Neck 36(5), 694–701 (2014). 23606370 10.1002/hed.23350 PMC4524549

[2] Gilbert MR, Sharma A, Schmitt NC A 20-year review of 75 cases of salivary duct carcinoma. JAMA Otolaryngol. Head Neck Surg. 142(5), 489–495 (2016). 26939990 10.1001/jamaoto.2015.3930 PMC5033043

[3] Johnston ML, Huang SH, Waldron JN Salivary duct carcinoma: treatment, outcomes, and patterns of failure. Head Neck 38(Suppl. 1), E820–E826 (2016). 25916947 10.1002/hed.24107

[4] Bakst RL, Su W, Ozbek U Adjuvant radiation for salivary gland malignancies is associated with improved survival: a National Cancer Database analysis. Adv. Radiat. Oncol. 2(2), 159–166 (2017). 28740927 10.1016/j.adro.2017.03.008 PMC5514258

[5] Xu B, Dogan S, Haroon Al Rasheed MR, Ghossein R, Katabi N. Androgen receptor immunohistochemistry in salivary duct carcinoma: a retrospective study of 188 cases focusing on tumoral heterogeneity and temporal concordance. Hum. Pathol. 93, 30–36 (2019). 31430492 10.1016/j.humpath.2019.08.007 PMC6937722

[6] Dalin MG, Desrichard A, Katabi N Comprehensive molecular characterization of salivary duct carcinoma reveals actionable targets and similarity to apocrine breast cancer. Clin. Cancer Res. 22(18), 4623–4633 (2016). 27103403 10.1158/1078-0432.CCR-16-0637 PMC5026550

[7] Dalin MG, Watson PA, Ho AL, Morris LG. Androgen receptor signaling in salivary gland cancer. Cancers 9(2), 17 (2017). 10.3390/cancers9020017 PMC5332940 28208703

[8] Sygut D, Bien S, Ziolkowska M, Sporny S. Immunohistochemical expression of androgen receptor in salivary gland cancers. Pol. J. Pathol. 59(4), 205–210 (2008). 19391487

[9] Yeoh CC, Dabab N, Rigby E Androgen receptor in salivary gland carcinoma: a review of an old marker as a possible new target. J. Oral Pathol. Med. 47(7), 691–695 (2018). 29863801 10.1111/jop.12741

[10] Van Boxtel W, Locati LD, van Engen-van Grunsven ACH, PALGA Group Adjuvant androgen deprivation therapy for poor-risk, androgen receptor-positive salivary duct carcinoma. Eur. J. Cancer 110, 62–70 (2019). 30771738 10.1016/j.ejca.2018.12.035

[11] Viscuse PV, Price KA, Garcia JJ, Schembri-Wismayer DJ, Chintakuntlawar AV. First line androgen deprivation therapy vs. chemotherapy for patients with androgen receptor positive recurrent or metastatic salivary gland carcinoma-a retrospective study. Front. Oncol. 9, 701 (2019). 31428578 10.3389/fonc.2019.00701 PMC6688187

[12] Boon E, van Boxtel W, Buter J Androgen deprivation therapy for androgen receptor-positive advanced salivary duct carcinoma: a nationwide case series of 35 patients in The Netherlands. Head Neck 40(3), 605–613 (2018). 29272069 10.1002/hed.25035 PMC5838735

[13] Corrêa TS, Matos GDR, Segura M, Dos Anjos CH. Second-line treatment of HER2-positive salivary gland tumor: ado-trastuzumab emtansine (T-DM1) after progression on trastuzumab. Case Rep. Oncol. 11(2), 252–257 (2018). 29867432 10.1159/000488669 PMC5981674

[14] Firwana B, Atassi B, Hasan R, Hasan N, Sukari A. Trastuzumab for Her2/neu-positive metastatic salivary gland carcinoma: case report and review of the literature. Avicenna J. Med. 2(3), 71–73 (2012). 23826550 10.4103/2231-0770.102282 PMC3697425

[15] Takahashi H, Tada Y, Saotome T Phase II trial of trastuzumab and docetaxel in patients with human epidermal growth factor receptor 2-positive salivary duct carcinoma. J. Clin. Oncol. 37(2), 125–134 (2019). 30452336 10.1200/JCO.18.00545

[16] Amini A, Waxweiler TV, Brower JV Association of adjuvant chemoradiotherapy vs radiotherapy alone with survival in patients with resected major salivary gland carcinoma: data from the National Cancer Data Base. JAMA Otolaryngol. Head Neck Surg. 142(11), 1100–1110 (2016). 27541166 10.1001/jamaoto.2016.2168

